# Rac2 is required for alternative macrophage activation and bleomycin induced pulmonary fibrosis; a macrophage autonomous phenotype

**DOI:** 10.1371/journal.pone.0182851

**Published:** 2017-08-17

**Authors:** Shweta Joshi, Alok R. Singh, Simon S. Wong, Muamera Zulcic, Min Jiang, Annie Pardo, Moises Selman, James S. Hagood, Donald L. Durden

**Affiliations:** 1 UCSD Department of Pediatrics, Moores UCSD Cancer Center, University of California, San Diego, United States of America; 2 Division of Respiratory Medicine, Department of Pediatrics, University of California, Rady Children's Hospital, San Diego, United States of America; 3 Facultad de Ciencias Universidad Nacional Autónoma de México Mexico City, Mexico; 4 Instituto Nacional de Enfermedades Respiratorias Ismael Cosío Villegas México Mexico City, Mexico; 5 Division of Pediatric Hematology-Oncology, UCSD Rady Children’s Hospital, San Diego, United States of America; Medical University of South Carolina, UNITED STATES

## Abstract

Idiopathic pulmonary fibrosis (IPF) is a chronic lung disease characterized by cellular phenotype alterations and deposition of extracellular matrix proteins. The alternative activation of macrophages in the lungs has been associated as a major factor promoting pulmonary fibrosis, however the mechanisms underlying this phenomenon are poorly understood. In the present study, we have defined a molecular mechanism by which signals transmitted from the extracellular matrix via the α_4_β_1_ integrin lead to the activation of Rac2 which regulates alternative macrophage differentiation, a signaling axis within the pulmonary macrophage compartment required for bleomycin induced pulmonary fibrosis. Mice deficient in Rac2 were protected against bleomycin-induced fibrosis and displayed diminished collagen deposition in association with lower expression of alternatively activated profibrotic macrophage markers. We have demonstrated a macrophage autonomous process by which the injection of M2 and not M1 macrophages restored the bleomycin induced pulmonary fibrosis susceptibility in Rac2-/- mice, establishing a critical role for a macrophage Rac2 signaling axis in the regulation of macrophage differentiation and lung fibrosis *in vivo*. We also demonstrate that markers of alternative macrophage activation are increased in patients with IPF. Taken together, these studies define an important role for an integrin-driven Rac2 signaling axis in macrophages, and reveal that Rac2 activation is required for polarization of macrophages towards a profibrotic phenotype and progression of pulmonary fibrosis *in vivo*.

## Introduction

Idiopathic pulmonary fibrosis (IPF) is a chronic and fatal disease characterized by fibrotic remodeling of lung parenchyma followed by progressive decline in lung function [1, 2]. IPF may be initiated by intrinsic and extrinsic cellular stress which lead to activation of fibroblasts, disruption of alveolar architecture, and deposition of extracellular matrix proteins, resulting in impaired gas exchange and respiratory failure [3–5]. The role of inflammation and immune dysregulation has been suggested as a major factor in the initiation and progression of pulmonary fibrosis [6].

Recent reports have highlighted that a discrete type of macrophage activation known as M2 or alternative activation is associated with the progression of IPF [7, 8]. Classical/M1 macrophage activation by Th1 cytokines and/or microbial agents, induces the production of interleukin 12 (IL12). Alternatively, macrophages stimulated by Th2 cytokines comprise the M2 phenotype which plays a critical role in wound healing and tumor progression [9]. M2 macrophages express higher levels of mannose receptor (MRC-1, CD206), and scavenging receptor CD163 as they differentiate from monocytes [10]. It is widely accepted that M2 terminology encompasses functionally diverse group of macrophages and can be further subdivided into M2a, M2b and M2c macrophages [9]. However, in reality, these *in vitro* concepts have been difficult to match to tissue macrophages *in situ*, due to lack of specific markers for these phenotypes within the tissue and numerous reports suggesting that macrophages exist as distinct entities *in situ* [11, 12]. Numerous studies point to the role of alternatively activated M2 macrophages in lung fibrosis in mouse models [7, 13–15] and in human IPF [8, 16]. M2 macrophages are capable of generating pro-fibrotic mediators, but this phenotype is ineffective to support the host defense role provided by the classically activated M1 macrophages [10]. In addition to a profibrotic role, this shift in macrophage phenotype in IPF patients may provide an explanation for why these patients get frequent lung infections, which leads to pulmonary failure and ultimately death [17]. Therefore, targeting the M2 macrophage phenotype is an attractive potential therapeutic strategy for lung fibrosis, as this phenotype produces high levels of growth factors and pro-fibrotic cytokines *in vitro*, which might contribute to the persistence of the fibrotic microenvironment. Although previous studies shed light on the molecular entities involved in alternative activation of MΘs [18–21], the critical signal transduction pathways that control the M1-M2 transition in lung fibrosis remain to be determined. If these pathways can be identified, they may serve as targets for therapeutic exploitation in lung fibrosis dependent upon M2 MΘs.

The role of Rac2, a small GTPase in hematopoietic and endothelial cell integrin and immunoreceptor signaling, is well documented [22–24]. Recently, our laboratory has shown that Rac2-/- mice are defective in tumor growth and the absence of Rac2 skews macrophages towards classical M1 activation [24]. Disruption of the gene encoding Rac2 led to attenuated lung inflammation and injury in a model of immune complex-mediated acute lung injury [25]. A small molecule inhibitor of Rac1 and Rac2 also reported to alleviate LPS induced acute lung injury [26]. A study by Arizmendi *et al* has shown that Rac2 promotes bleomycin induced lung injury, however the molecular mechanism remains unclear [27]. In the present study, we investigated the mechanism by which Rac2 regulates fibrogenesis in a bleomycin induced model of pulmonary fibrosis. Our data demonstrate a novel signaling pathway within the lung fibrotic milieu downstream of the extracellular matrix and the α_4_β_1_ integrin in which Rac2 is necessary and sufficient to control alternative macrophage activation in the setting of bleomycin induced lung injury. We suggest that further analysis of the genetic and/or epigenetic alterations observed in the Rac2 -/- model which abrogate fibrosis and the M2 phenotype may contribute to understanding the process of lung fibrogenesis observed in IPF and suggest new targets for therapeutic exploitation.

## Materials and methods

### Animals

Rac2-/- mice (backcrossed >50 generations into C57BL/6J background) and genotyping have been previously described [23]. Integrin α4Y991A mice were a gift from Dr. Mark Ginsberg [28]. α4Y991A mice bears a point mutation in α4 integrin cytoplasmic tail (Y991A) and are reported to show a deficit in recruitment of leukocytes to inflammatory sites with no defect in recruitment of neutrophils. For our experiments, we used female animals at 8–10 weeks of age. C57BL/6 animals were used as controls. All procedures involving animals were approved by the University of California San Diego Animal Care Committee, which serves to ensure that all federal guidelines concerning animal experimentation are met.

### Bleomycin induced pulmonary fibrosis

WT, Rac2-/- and α4Y991A mice were anesthetized with intraperitoneal injections of ketamine and xylazine (100 and 10 mg/kg body mass, respectively). Mice were administered an intratracheal instillation of 0.05 U of bleomycin sulphate (Sigma) (3 U/Kg body weight, dissolved in normal saline) or normal saline using Micro Sprayer MS-IA-1C (Penn-Century). Following the bleomycin instillation, mice were monitored daily for morbidity and mortality. Groups of WT, Rac2-/- and α4Y991A mice (*n* = 5 to 10 per time point) were sacrificed and their lung tissues were analyzed on day 3, 7 and 28 after bleomycin injection.

### Lung histology and immunohistochemistry

For murine lung histology, control and bleomycin instilled WT, Rac2-/- and α4Y991A were euthanized on day 28. The thoracic cavity was dissected to expose the lungs, tracheae were cannulated, and 1.0–1.5 ml of 10% buffered formalin in PBS (pH 7.2) was gently instilled by syringe through the cannula into the lungs. Lungs were inflated to the original dimensions of the thoracic cavity. After instillation of buffered formalin, lungs were stored in 10% buffered formaldehyde for 24 h and then processed for histological studies. For OCT embedding, the lungs were inflated similarly with OCT sucrose mixture and stored at -80°C. Lungs were sectioned at 4 μm followed by hematoxylin and eosin (H&E) staining or Sirius red staining, or with antibodies for α-smooth muscle actin (αSMA).

### Hydroxyproline assay

Lung samples from WT, Rac2-/- and α4Y991A mice at day 28 after bleomycin instillation were removed and stored at -80°C for hydroxyproline assay as described previously [29] with some modifications. The left lung from each mouse was hydrolyzed for 12 h in 6N HCL at 110°C. The hydrolysate was then separated by centrifugation and 20 μl of each sample was then incubated with 100 μl Chloramine-T solution [0.564g chloramine-T was dissolved in 32mL Citrate/Acetate Buffer and 4ml n-propanol] at room temperature for 15 min, followed by incubation with 100 μl Ehrlich's Solution [4.5g DMAB in 18.6 mL n-propanol and 7.8 mL perchloric acid] at 65°C for 10 min. Absorbance was read at 550 nm and hydroxyproline concentrations were calculated from standard curve generated using known concentrations of trans-4- hydroxyl-L-proline (Sigma).

### Broncho alveolar lavage

Briefly, the mice were euthanized on day 3, 7 and 28 after i.t. bleomycin instillation, and the trachea was exposed in a sterile manner. The lungs were lavaged 4 times with 1 mL of phosphate-buffered saline (PBS), and the lavage was withdrawn from the lungs with a 0.8–0.9 mL volume return. The lavage fluids were pooled and centrifuged at 1000 x *g* for 10 min at 4°C, followed by resuspension of cell pellets in 1mL of PBS. BAL fluid was centrifuged at 300 *x* g for 15 min, total cell counts were determined using hemocytometer and 5000 cells were used to prepare cell cytospins. Differential Diff quick Stain kit (Electron Microscopy Sciences, PA, USA) was used to differentiate macrophages, neutrophils and lymphocytes. BAL supernatants were stored at -80°C and were used for measuring albumin using Pierce BSA kit (Thermo Fisher Scientific, Rockford, IL).

### Bone marrow derived macrophages (BMDMs) and BAL macrophage culture

Bone marrow-derived macrophages (BMDM) were isolated as described previously [24]. M2 and M1 macrophages were generated by stimulating BMDMs with either 20 ng/ml of IL4 or 100 ng/ml of LPS. Alveolar macrophages were obtained from the BALs of WT, Rac2-/- and α4Y991A mice on day 3, 7 and 28 after intrapulmonary bleomycin challenge. BAL cells were plated in 10 cm tissue culture plates at a density of 1.0 x 10^6^ cells/ml in RPMI plus 10% fetal calf serum and incubated for 2 hour at 37°C. Nonadherent cells were discarded and adherent alveolar macrophages were subjected to RNA isolation or Western Blot analysis.

### Western blots

Alveolar macrophages were washed with PBS and lysed with 1X RIPA buffer containing protease inhibitor cocktail (Thermo Scientific). Fifty micrograms of protein lysates were resolved by SDS-PAGE. Membranes were incubated with antibodies against FIZZ1 (Abcam), Arg (BD Biosciences) and CD206 (Abcam) antibodies. Signals were developed with Pierce ECL Western blot detection kit (Thermo Scientific, Rockford, IL).

### Arginase activity

Arginase activity was measured in alveolar macrophages isolated from BALs on day 28, as previously described [24]. Briefly, macrophages were lysed with 100 μl of 0.1% Triton X-100, followed by addition of 100 μl of 50 mM Tris-HCl and 10 μl of 10 mM MnCl_2_ and heating for 10 min at 56°C. Arginine was hydrolyzed by incubating the lysate with 100 μl of 0.5 M L-arginine (pH 9.7) at 37°C for 60 min. The reaction was terminated with 400 μl of H_2_SO_4_ (96%)/H_3_PO_4_ (85%)/H_2_O (1/3/7, v/v/v) mixture. Absorbance was read at 540 nm after addition of 25 μl of α-isonitrosopropiophenone, and heating of samples at 95°C for 45 min. One unit of enzyme activity is defined as the amount of enzyme that catalyzes the formation of 1 μmol urea per min.

### Quantification of gene expression

Total RNA was isolated from BALs using the Qiagen RNAeasy kit (Qiagen, Hilden, Germany) according to manufacturer’s protocol. Then 1 μg RNA was reverse transcribed in 20 μl volume using iscript cDNA synthesis kit (Bio-Rad, Hercules, CA). Messenger RNA expression was detected in 2 μl of cDNA in 96-well plates on an CFX96 Real time system (Bio-Rad, Hercules, CA) using 1× SYBR green supermix (Bio-Rad, Hercules, CA). The program used was: 5 min at 95°C, and then 40 cycles of 20 s at 95°C, 1 min at 58°C and 30 sec at 72°C. The primers used for murine IL1, IL6, TNF alpha, YM1, CD206, Fizz1 and arginase were previously described [24]. Relative expression levels were normalized to Gapdh expression and values are multiplied by 100 for presentation purposes.

### Macrophage injection experiments

To determine if the phenotypes observed in the Rac2-/- mice were macrophage autonomous, we reconstituted Rac2-/- mice with bone marrow derived macrophages from wild type mice. Rac2-/- mice were administered a single intratracheal injection of 0.05 U of bleomycin sulphate (3 U/Kg body weight, dissolved in normal saline, Sigma Aldrich) or normal saline on day 0. On 5^th^ day following bleomycin instillation, 1 x 10^6^ cultured WT M2 or M1 BMDMs (M2 macrophages were BMDMs stimulated with 20 ng/ml of IL4 and M1 macrophages stimulated with 100 ng/ml of LPS) were injected through the tail vein in Rac2-/- mice. 1 million M2 or M1 macrophages were injected every third day until lungs were harvested on 28 day.

### Human patient samples

BAL pellets from patients diagnosed with IPF or hypersensitivity pneumonitis [30], diagnosed according to published consensus statements were received as 1 ml frozen pellets. The local ethics committee of the Instituto Nacional de Enfremedades Respiratorias (INER, Mexico City, Mexico) approved the human BAL study. These samples were revived in RPMI +10% FBS and centrifuged at 1100 rpm for 10 min, followed by RNA isolation and RTPCR as described.

### Statistical analysis

Values are expressed as mean ± SEM. Statistical differences in the mean values among treatment groups were determined by using a one-way ANOVA test with posthoc analysis using Tukey’s multiple comparison test. In all cases, a value for p < 0.05 was considered statistically significant.

## Results

### Rac2 is required for bleomycin induced lung fibrosis

**Fig 1:**
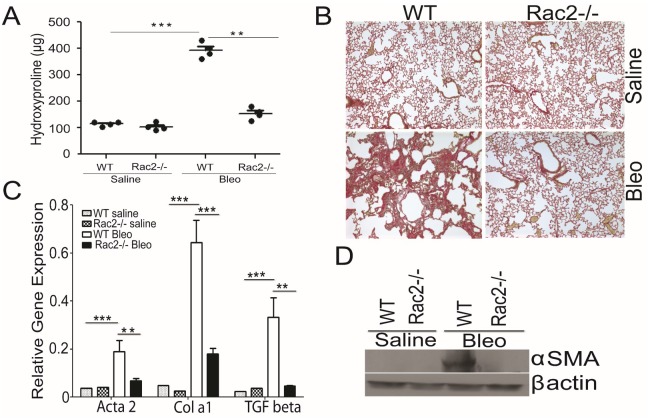
Rac2 is required for bleomycin-induced pulmonary fibrosis. WT and Rac2-/- mice (n = 7–8 mice/group) were given an i.t. challenge with bleomycin or saline, and whole lungs were isolated on day 28. (A) Hydroxyproline assay showing the levels of collagen in whole lungs (n = 4 mice/ group). (B) mRNA expression of fibrosis related genes in whole lungs (n = 3). (C) Sirius red staining of histological sections. Original magnification 20X (D) Western blot of Smooth muscle actin performed on whole lungs from n = 1 mouse / group. Graphs in A and C represent mean ± SEM with n = 3–4 samples/group. Data was analyzed by One-way ANOVA with post-hoc Tukey’s multiple comparison tests, **p ≤ 0.01 and ***p ≤ 0.001 when WT bleomycin treated group was compared to saline treated groups or the Rac2-/- bleomycin treated group. Data shown here is representative of one experiment, but all experiments were repeated three times with similar results.

Rac2 is expressed primarily in hematopoietic cells, and plays an important role in actin cytoskeletal rearrangements, superoxide production and degranulation in neutrophils and macrophages [31, 32]. A previous study has shown that Rac2 mediates acute lung injury in a murine model [25]. A recent study by Arizmendi *et al* has shown that Rac2 null mice are protected against bleomycin lung injury [27] however the molecular mechanism regulating this phenomenon remains unclear. Moreover, this study did not report alterations in lung fibrosis or any link to macrophage biology between the two experimental groups. To determine whether Rac2 was critical for the development of bleomycin-induced pulmonary fibrosis, WT and Rac2-/- mice were challenged with 3 U/Kg of bleomycin and the amount of fibrosis was measured. Administration of bleomycin by the intratracheal route induces initial inflammation and subsequent fibrosis. Fibrosis begins to develop by 14 days and usually peaks between 21–28 days [33]. In our model, administration of bleomycin (3U/kg), in WT C57BL/6 mice reliably cause fibrosis in 28 days (Fig 1). Saline 0.9% does not induce fibrosis (Fig 1). To quantitate fibrosis, whole lungs were harvested from WT and Rac2-/- on day 28 after bleomycin and the amount of collagen was measured by hydroxyproline assay. Whole lung samples from saline treated Rac2-/- mice exhibited similar hydroxyproline levels to those observed in WT saline. WT Bleo mice had significantly higher collagen deposition on day 28 after bleomycin challenge, as compared with saline treated mice and Rac2-/- Bleo mice, (Fig 1A). Sirius red staining in Fig 1B showed that WT and Rac2-/- saline group are similar in histological architecture. As expected, lungs of bleomycin-treated WT mice showed extensive disruption of lung architecture, obstruction of alveolar spaces, and an extended web of collagen-positive stained areas in an irregular pattern (Fig 1B). In contrast, lungs of Rac2-/- mice treated with i.t. bleomycin showed a normal histopathological lung architecture and less collagen accumulation within the lung (Fig 1B). Moreover, quantitation of expression of the fibrosis marker genes in the lung parenchyma of Rac2-/- vs WT mice allowed us to correlate the lung fibrotic phenotype with the augmented expression of a M2 pro-fibrotic gene signature (Fig 1C). These results were further supported by increased α-SMA staining in WT bleomycin treated group as compared to Rac2-/- as revealed by Western blotting (Fig 1D). These data strongly support our conclusion that the deletion of the Rac2 gene in mice prevents bleomycin-induced pulmonary fibrosis.

In order to investigate the role of Rac2 in bleomycin-induced lung injury, lung physiology was analyzed in the bleomycin instilled Rac2-/- vs WT mice on day 28. WT mice that received i.t. bleomycin showed significantly augmented airway resistance and elastance compared to saline treated mice (S1 Fig). Similar to previously published report [27] Rac2−/− mice that received bleomycin showed resistance and elastance levels that were similar to Rac2−/− or WT mice receiving saline. There was a highly significant difference in resistance and elastance between bleomycin-treated WT and Rac2−/− mice. (S1 Fig). To further understand the function of Rac2 in the long-term effects of bleomycin-induced lung injury, WT and Rac2−/− mice challenged with bleomycin were monitored for mortality up to day 28. We did not observe any mortality in WT mice by day 28. Taken together, the fibrosis and pulmonary physiology results indicate that bleomycin-induced lung pathology is markedly attenuated in Rac2−/− mice.

### Impaired alternative activation of macrophages is associated with reduced fibrosis in Rac2-/- following bleomycin

**Fig 2:**
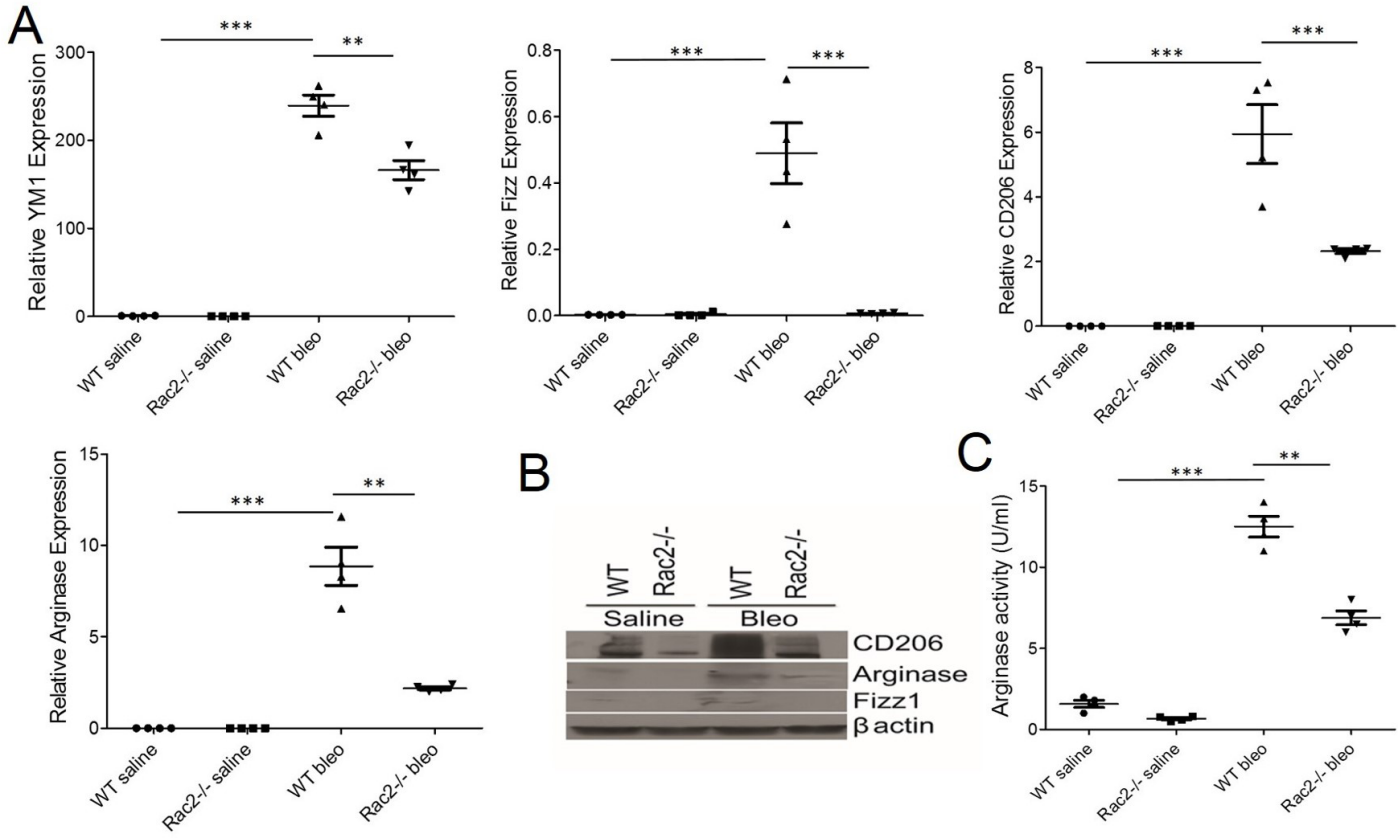
Rac2 is required for enhanced alternative activation of the macrophage phenotype after bleomycin-induced pulmonary fibrosis. (A & B) WT and Rac2-/- mice were given an i.t. challenge with bleomycin and alveolar macrophages were isolated from BALs on day 28 (n = 8–10 mice/group). All the BAL samples/group were pooled to isolate alveolar macrophages and were used for mRNA expression or Western blot or arginase activity. The expression of alternatively activated markers YM1, Fizz1, CD206 and Arginase in alveolar macrophages were analyzed by real-time RT-PCR (A) or Western blot (B). (C) Arginase activity was measured in alveolar macrophages isolated from BALs from bleomycin instilled WT and Rac2-/- mice on day 28 as described in Methods. Graphs in A and C represent mean ± SEM with n = 4. One-way ANOVA with post-hoc Tukey’s multiple comparison tests, **p ≤ 0.01 and ***p ≤ 0.001 when WT bleomycin treated group was compared to saline treated groups or the Rac2-/- bleomycin treated group. Experiment was repeated twice with similar results.

Our results clearly establish a requirement for Rac2 in bleomycin induced lung fibrosis. The next question we addressed experimentally was; how does this small GTPase, which is specifically expressed in the hematopoietic compartment, modulate fibrosis and the alterations in macrophage polarization in the injured lung? One explanation for the Rac2-/- phenotype is that Rac2 is required for promoting alternative activation of M2 macrophages in the lung parenchyma following lung injury. In a recent study, we reported that Rac2-/- is required for tumor growth and M2 macrophage differentiation in tumor microenvironment [24]. Our previously published genomic and metabolomics results in cancer models suggested that Rac2-/- BMDMs are defective in M2 macrophage differentiation *in vitro* and *in vivo* [24]. On the basis of the literature suggesting role of M2 macrophages in fibrosis [7, 8] and role of Rac2 in M2 macrophage differentiation [24] we hypothesized that Rac2-/- mice were protected from fibrosis due to a defect in alternative activation of M2 macrophages within the injured lung. To test this hypothesis, we used RT-PCR to detect the expression of M2 markers in the alveolar macrophages isolated from the BAL of bleomycin instilled WT and Rac2-/-, animals on day 28. We found that BAL derived MΘs isolated from Rac2-/- bleomycin treated mice were defective in the differentiation of macrophages into M2 phenotype as shown by RT-PCR analysis (Fig 2A). Macrophages isolated from BALs of bleomycin instilled Rac2-/- mice displayed a significant downregulation in gene expression of typical M2 markers viz., YM1, Fizz1, CD206 and arginase (Fig 2A). Importantly, both WT and Rac2-/- saline treated experimental groups did not increase the expression of these M2 genes. These results were further validated by Western blot analysis showing reduced expression of CD206, arginase and Fizz 1 in alveolar MΘs isolated from bleomycin instilled Rac2-/- mice (Fig 2B). Consistent with these data, we observed augmented arginase activity in BAL-associated MΘs isolated from WT mice instilled with bleomycin, a result not seen in Rac2-/- BAL-associated pulmonary MΘs (Fig 2C).

### Rac2-/- mice display inflammatory M1 phenotype with impaired M2 macrophage activation in late phase of bleomycin induced lung injury

**Fig 3:**
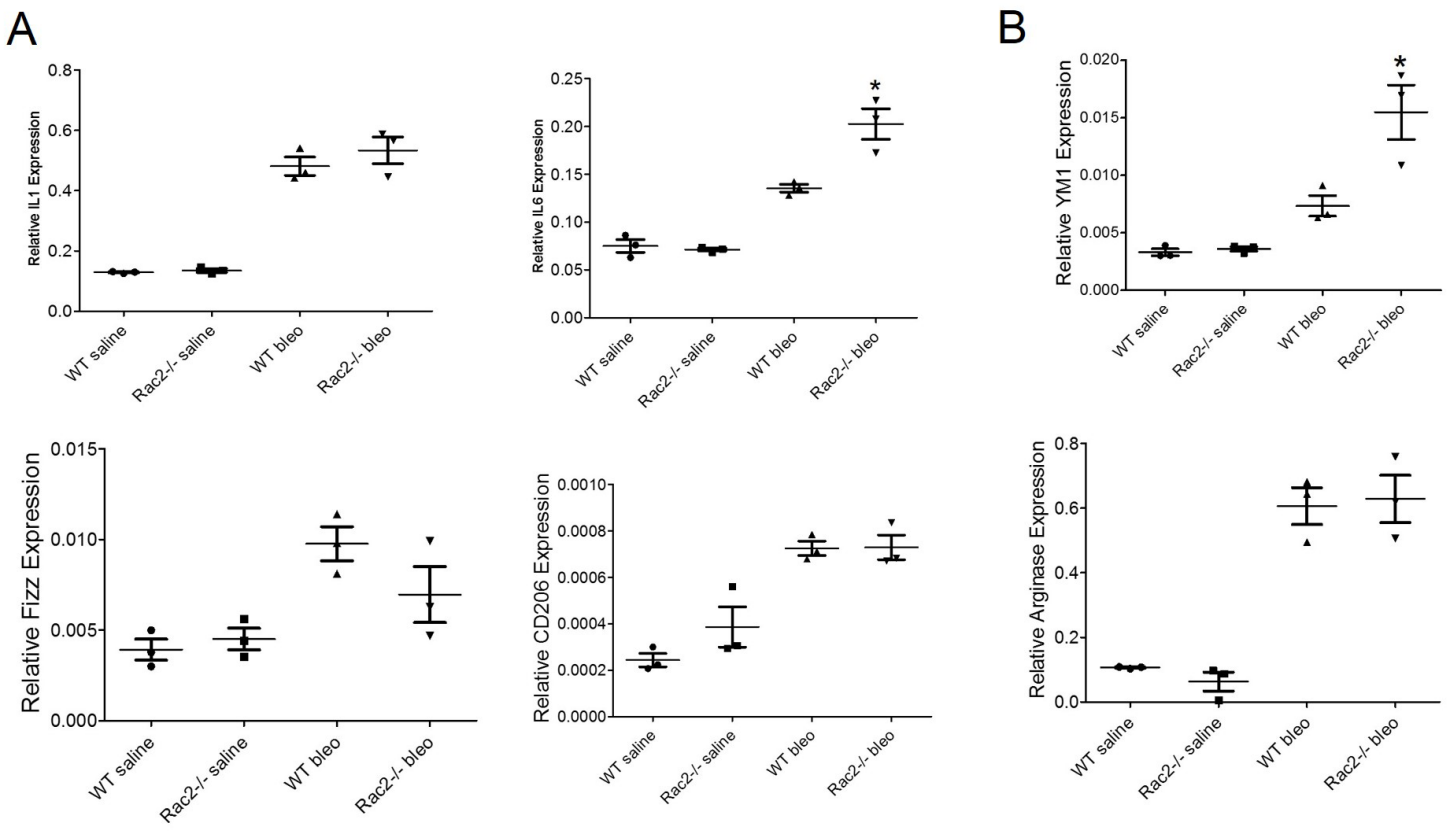
Rac2-/- mice display augmented expression of inflammatory M1 macrophage markers in early phase of bleomycin induced lung injury. WT and Rac2-/- mice (n = 8–10 mice/ group) were given an i.t. challenge with bleomycin or saline, and whole lungs or alveolar macrophages from BALs were isolated on day 3. BAL samples from 2–3 mice were pooled together to isolate alveolar macrophages and were used for mRNA expression (A & B). Expression of M1 (A) and M2 (B) marker genes in the alveolar macrophages isolated from BALs of WT and Rac2-./- mice challenged with bleomycin on day 3. Graphs represent mean ± SEM with n = 3 pooled samples. *p ≤ 0.05 when WT bleomycin treated group was compared with Rac2-/- bleomycin treated group.

**Fig 4:**
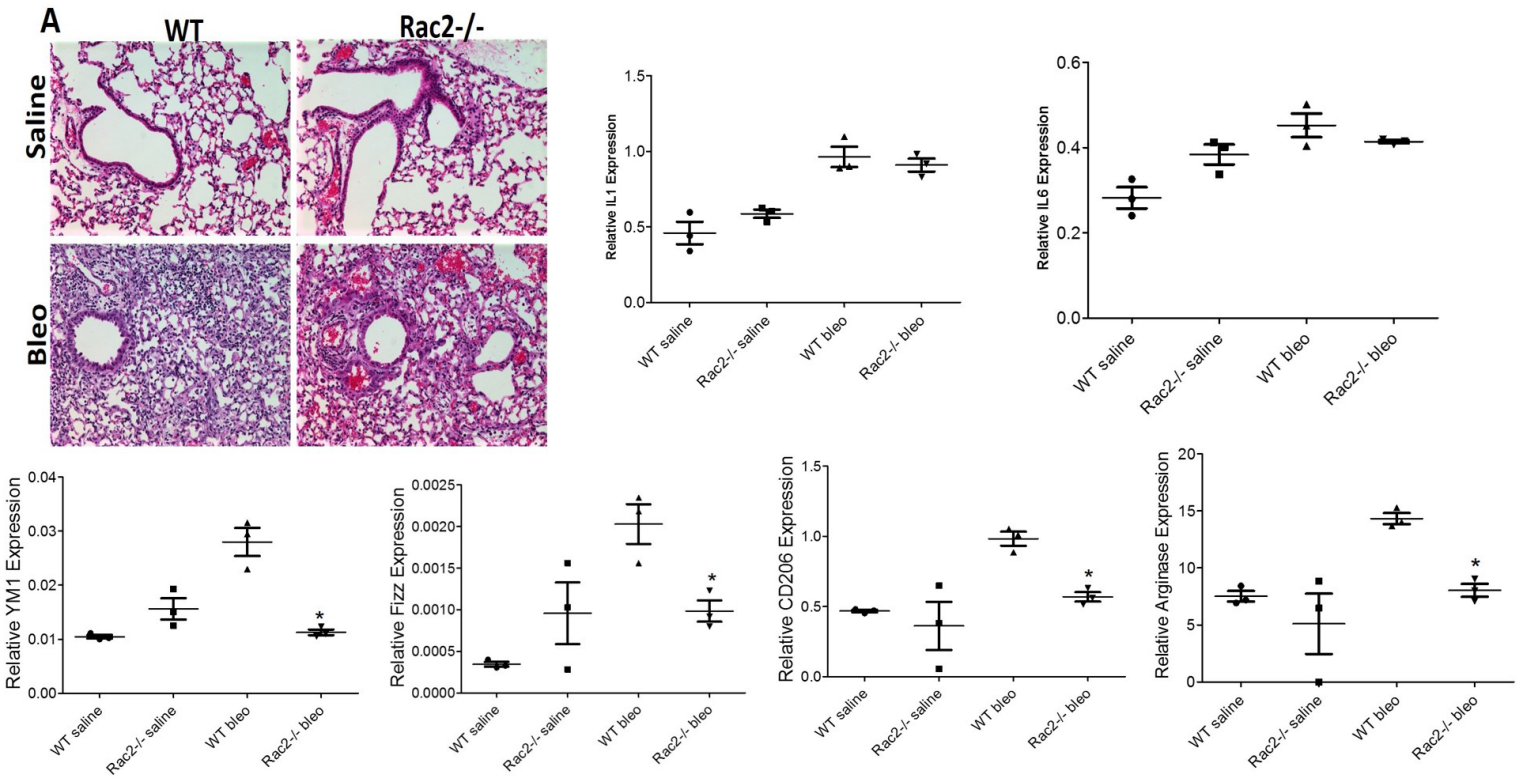
Massive infiltration of leukocytes in lungs of WT and Rac2-/- mice challenged with bleo on day 7 with impaired activation of M2 macrophages. (A) H & E staining of the whole lungs isolated on day 7 showing massive infiltration of leukocytes in bleomycin instilled WT and Rac2-/- mice (n = 8 mice / group). (B & C). BAL samples from 2–3 mice were pooled together to isolate alveolar macrophages and were used for mRNA expression. Expression of M1 (B) and M2 (C) marker genes in the alveolar macrophages isolated from BALs of WT and Rac2-./- mice challenged with bleomycin on day 7. Graphs represent mean ± SEM with n = 3 pooled samples. *p ≤ 0.05 when WT bleomycin treated group was compared with Rac2-/- bleomycin treated group.

The data presented above clearly demonstrate that Rac2-/- mice display a defect in alternative activation of M2 macrophage phenotype in bleomycin instilled mice on day 28. This observation led us to investigate the modulation of macrophage phenotypes in the early phase of lung injury in Rac2-/- mice. In order to get clear picture of how Rac2 controls early phase of lung injury, we determined the extent of early lung injury and inflammation in bleomycin instilled WT and Rac2-/- mice on day 3 and 7. There were no observed differences in BAL cell counts or albumin (indicative of vascular leak [34]) between WT and Rac2-/- mice at day 3 (S2 and S3 Figs). Similar to a previous report [27], we also observed that neutrophils were reduced in bleomycin instilled Rac2-/- mice compared to WT mice on day 7, but there was no significant difference in the no. of macrophages or lymphocytes infiltrated (S2 Fig). Albumin levels in bleomycin instilled Rac2-/- mice were also lower compared to WT on day 7 (S3 Fig), consistent with a role of Rac2 in neutrophil-mediated transition from early to later stages of acute lung injury. Consistent with a previous report [27] our data indicate (S2 Fig) that macrophage infiltration is normal in Rac2 -/- mice instilled with bleomycin on day 3 and 7. We next measured Cola1 (collagen) gene expression on days 7, 14 and 28 following bleomycin challenge. Our results showed that at day 7 there is no statistically significant increase in collagen in either genotype. However in the fibroproliferative phase at day 14 increasing collagen gene expression is manifest, with statistically higher levels in WT (p < .05). (S4 Fig). Importantly, a statistically greater level of collagen expression is noted on day 28 (P < .001) in WT vs Rac2-/-. The results from the time course analysis of collagen expression provides indicates that the most significant differences in fibrosis in this model occur in the later stages. These results prompted us to examine if there is any modulation in macrophage phenotypes in bleomycin challenged WT and Rac2-/- mice in the transition from acute injury to fibrosis. Fig 3A illustrates that macrophages derived from BALs of bleomycin instilled WT and Rac2-/- mice display increased expression of inflammatory M1 macrophage genes viz. IL1 and IL6 in both the genotypes, with significant upregulation of IL6 in Rac2-/- mice on day 3. The expression of M2 marker genes viz. YM1, Fizz1, CD206 and arginase is similar in both WT and Rac2-/- on day 3, except YM1, which shows significant upregulation in the Rac2-/- bleomycin experimental group. It is important to state, that the expression of M2 genes is low compared to day 28 (Fig 3B), suggesting that in early phase of lung injury, macrophages are predominantly of inflammatory M1 phenotype. Fig 4A shows massive infiltration of leukocytes into the lungs of both WT and Rac2-/- mice instilled with bleomycin on day 7 with no disruption of normal lung architecture in both genotypes. The expression of inflammatory M1 genes is high in both genotypes (Fig 4B). Interestingly, the expression of anti-inflammatory M2 genes is high in WT mice instilled with bleomycin on day 7 when compared to Rac2-/- mice (Fig 4C) and WT mice instilled with bleomycin on day 3 (Fig 3B), implicating the shift of pro inflammatory M1 macrophages to anti-inflammatory M2 macrophages in WT mice on day 7. These findings indicate that the absence of fibrosis at day 28 in Rac2-/- mice is not simply due to protection from acute lung injury, but rather suggest that a Rac2-macrophage autonomous event regulates the alternative activation of MΘ phenotype and progression to lung fibrosis *in vivo*.

### α_4_β_1_ integrin activate Rac2 and promotes pulmonary fibrosis

**Fig 5:**
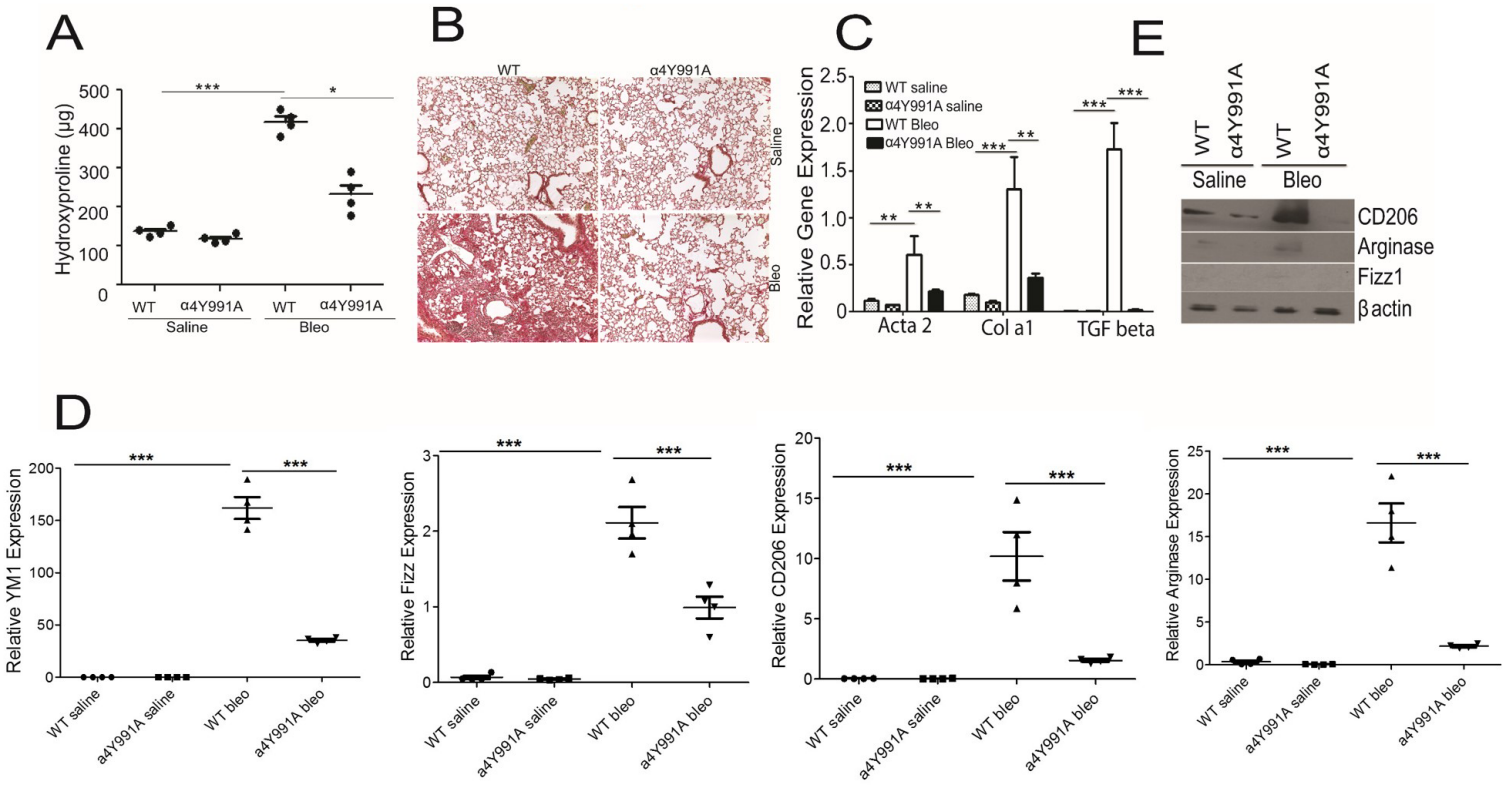
α_4_β_1_ integrin is required for bleomycin-induced pulmonary fibrosis. (A) WT and α4Y991A mice were given an i.t. challenge with bleomycin (n = 8 mice / group), and whole lungs were isolated on day 28 and amount of collagen was assessed by hydroxyproline assay (n = 4 mice/group) (A), Sirius red staining of histological sections, Original magnification 20X (B), mRNA expression of fibrosis related genes (C) in saline and bleomycin instilled lungs isolated from WT and α4Y991A mice (n = 3 samples/group). For mRNA analysis and Western blot, BAL samples from n = 8 mice / group were pooled together to isolate alveolar macrophages for either mRNA expression or Western blot. (D) Expression of alternatively activated markers YM1, Fizz1, CD206 and Arginase in alveolar macrophages isolated from BALs from bleomycin instilled WT and α4Y991A mice on day 28 and analyzed by real-time RT-PCR. (E) Western blot analysis of M2 marker genes in bleomycin challenged WT and α4Y991A mice. Graphs represent mean ± SEM with n = 4 samples for A, n = 3 for C and n = 4 for D. One-way ANOVA with post-hoc Tukey’s multiple comparison tests, *p ≤ 0.05, ***p ≤ 0.001 when WT bleomycin treated group was compared to saline treated groups or the Rac2-/- bleomycin treated group. Experiment was repeated twice with same results.

Our published results clearly establish that the α_4_β_1_ integrin is a potent selective upstream stimulus for Rac2 activation in macrophages. The α_4_β_1_ integrin promotes Rac2 activation, macrophage migration and M2 macrophage differentiation [24]. Our recently published results [24] showed less Rac2-GTP activation under conditions of CSF1R/α_4_β_1_ engagement in α4Y991A knock-in mice as compared to WT mice. These observations lead us to examine the role of α_4_β_1_ specific integrin in pulmonary fibrosis and M2 macrophage differentiation in a setting of bleomycin induced pulmonary injury. To determine this α4Y991A k/in mice were instilled intratracheally with 3 U/Kg of bleomycin intratracheally and subsequently examined for fibrosis and macrophage differentiation on day 28. It is reported that mice homozygous for α4 integrin mutation [α4(Y991A)] are deficient in paxillin binding and manifest a profound deficit in the recruitment of mononuclear leukocytes to an inflammatory site with no defect in neutrophil recruitment [28]. Fig 5A summarizes the whole lung hydroxyproline levels detected on day 28 after bleomycin challenge. Whole lung samples from saline treated α4Y991A k/in mice exhibited similar hydroxyproline levels to those observed in WT saline. More importantly, hydroxyproline levels were significantly lower in bleomycin instilled α4Y991A k/in lungs compared with WT bleo group (Fig 5A). Sirius red staining in Fig 5B shows that WT and α4Y991A k/in saline group did not differ histologically. In contrast, a comparison of whole lung samples from bleomycin instilled WT and α4Y991A k/in mice revealed an increased extracellular deposition of collagen in WT as compared to α4Y991A k/in mice (Fig 5B). We also observed increase in the induction of Acta 2, Cola1 and TGF beta in WT bleo group as compared to α4Y991A k/in bleomycin treated mice on day 28 (Fig 5C). These results suggest that α_4_β_1_ integrin promotes bleomycin induced pulmonary fibrosis. Based on our previous report showing that α4Y991A k/in mice are defective in M2 macrophage differentiation [24], we next investigated if resistance of α4Y991A k/in mice to bleomycin induced lung fibrosis was associated with defects in alternative activation of M2 macrophages. Fig 5D shows the RTPCR data illustrating the significant reduction in gene expression of M2 macrophage markers viz., YM1, Fizz1, CD206 and arginase in MΘs isolated from BALs of bleomycin treated α4Y991A mice on day 28. These results were further validated by Western blot analysis (Fig 5E). Importantly, based on our previous published report [24] and the results generated in the present study we conclude that α_4_β_1_-dependent Rac2 activation in macrophages controls the transcription regulation of M2 macrophage differentiation in setting of bleomycin induced lung injury and pulmonary fibrosis.

### Macrophage autonomous, M2 dependent nature of Rac2 defect in promotion of bleomycin induced pulmonary fibrosis

**Fig 6:**
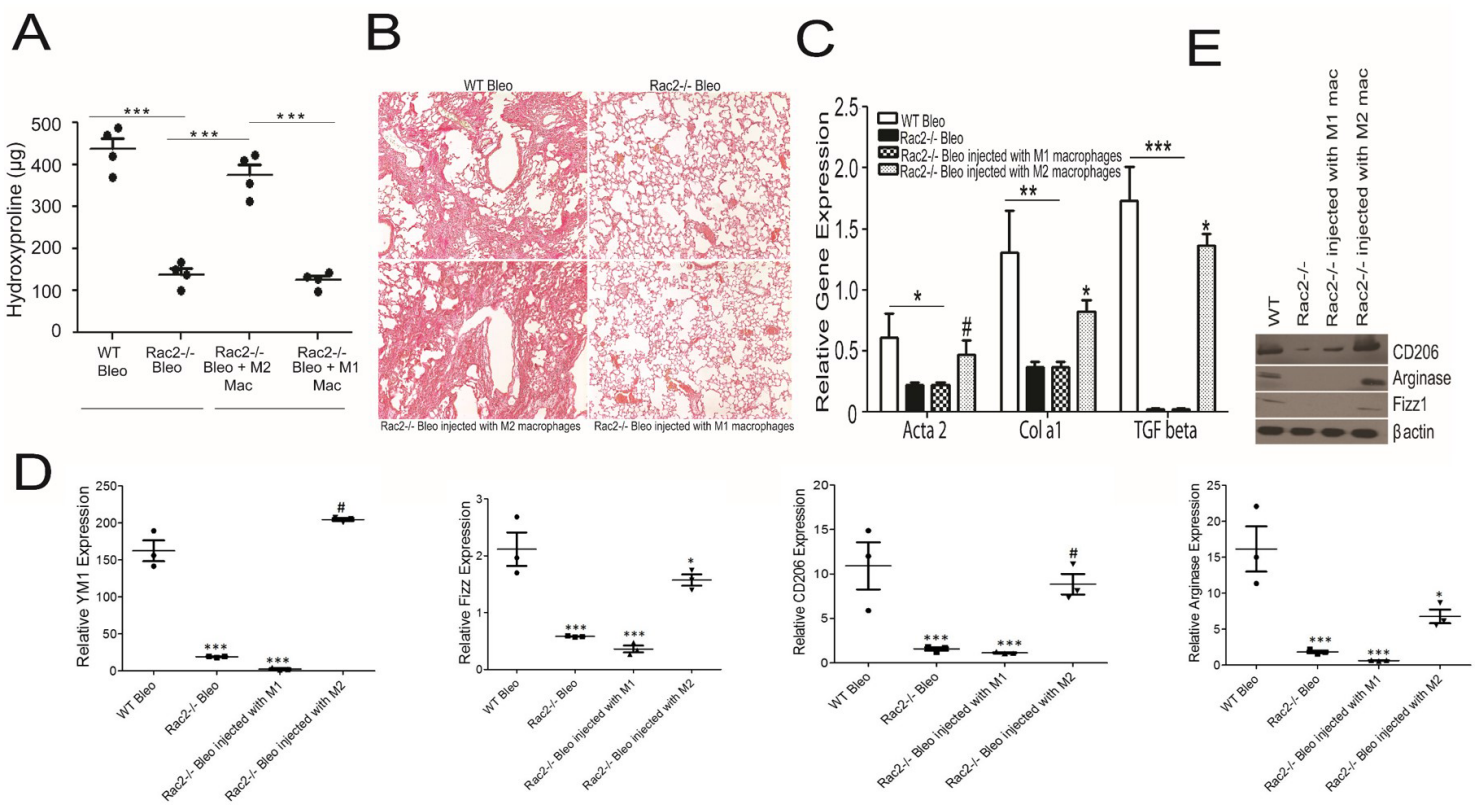
Reversal of the pulmonary fibrosis defect in Rac2-/- mice by injection of M2 macrophages. WT and Rac2-/- mice were given an i.t. challenge with bleomycin (n = 6 mice / group) and 5 days after bleomycin instillation, Rac2-/- mice were treated with 1 X 10^6^ M1 or M2 macrophages (generated by stimulation of 7 day old MCSF grown macrophages stimulated with 50 ng/ml of LPS or 20 ng/ml of IL4 for 20 hrs respectively), twice a week by tail vein injections until lungs were harvested on day 28. (A) Hydroxyproline assay showing levels of collagen in WT, Rac2-/- mice treated with or without M1 and M2 macrophages (n = 4 mice/group). (B) shows Sirius red staining of lungs isolated from 3U/kg bleomycin instilled WT and Rac2-/- mice injected with or without M1 or M2 macrophages. (C) shows mRNA expression of fibrosis related genes in whole lungs (n = 3) isolated from bleomycin instilled WT and Rac2-/- mice injected with or without M1 or M2 macrophages. For mRNA analysis and Western blot, BAL samples from n = 6 mice / group were pooled together to isolate alveolar macrophages for mRNA expression or Western blot. (D) Gene expression of M2 macrophage markers in alveolar macrophages isolated from BALs from bleomycin instilled WT and Rac2-/- mice injected with or without M1 or M2 macrophages (E) Western blot analysis of M2 marker genes in alveolar macrophages isolated from BALs from bleomycin instilled WT and Rac2-/- mice injected with or without M1 or M2 macrophages. Graphs represent mean ± SEM with n = 4 samples in A and n = 3 sample in C. Data was analyzed by One-way ANOVA with post-hoc Tukey’s multiple comparison tests, *p ≤ 0.05, **p ≤ 0.01, ***p ≤ 0.001, # not significant when WT bleomycin treated group was compared to Rac2-/- bleo group, injected with or without M1 and M2 macrophages.

Since Rac2 is expressed in a number of hematopoietic lineages and in endothelial cells [35], we sought to determine if the pulmonary fibrosis defect noted in the Rac2-/- mouse model could be ascribed to a macrophage autonomous M2 dependent phenomenon. If so, we would predict that the injection of WT M2 macrophages and not WT M1 macrophages into Rac2-/- mice would restore the bleomycin induced pulmonary fibrosis susceptibility and rescue the lung fibrosis defect observed in the Rac2-/- model *in vivo*. This experiment would provide evidence that the profibrotic defect in the Rac2-/- mouse model is mechanistically linked to the M1-M2 transition defect in the macrophage lineage. Previously, we reported this methodology to determine whether a defect in Rac2 was macrophage autonomous and M2 dependent in the setting of metastasis [24]. M1 and M2 macrophages were generated by stimulating bone marrow derived macrophages (BMDMs) by LPS and IL4 respectively and were tested for specific gene expression before injections (S5 Fig). As predicted, the injection of WT M2 BMDMs and not WT M1 BMDMs increased fibrosis after lung injury (Fig 6). Fig 6A shows the whole lung hydroxyproline levels detected on day 28 after bleomycin challenge. Whole lung samples from bleomycin treated WT mice exhibited similar hydroxyproline levels to those observed in Rac2-/- bleomycin mice treated with M2 macrophages. More importantly, hydroxyproline levels were similar in bleomycin instilled Rac2-/- lungs compared Rac2-/- bleo mice treated with M1 macrophages (Fig 6A). Sirius red staining demonstrated that Rac2-/- bleomycin treated mice had similar lung architecture as the Rac2-/- bleomycin treated mice injected with M1 macrophages. Rac2-/- bleomycin treated mice injected with M2 macrophages on the other hand, showed extensive collagen deposition, disruption of lung architecture as observed in WT bleomycin (Fig 6B). Consistent with histopathology, the expression of fibrosis markers is significantly reduced in the Rac2-/- bleomycin treated and mice injected with M1 macrophages as compared to WT bleomycin treated animals. In contrast, the Rac2-/- bleomycin treated mice injected with M2 macrophages demonstrated an upregulation of all the fibrosis genes as compared to Rac2-/- bleomycin treated mice treated with injection of M1 MΘs (Fig 6C). Moreover, the injection of M2 macrophages resulted in an increased alternative activation of M2 macrophages as demonstrated by expression of YM1, Fizz1, CD206 and arginase in macrophages isolated from BALs as compared to Rac2-/- bleomycin treated and the Rac2-/- bleomycin treated mice injected with M1 macrophages (Fig 6D). Notably, the expression of YM1 and CD206 is similar to the WT bleomycin treated mice (Fig 6D). Similar to this, Western blot analysis also showed higher expression of M2 proteins in Rac2-/- bleomycin mice challenged with M2 macrophages (Fig 6E). Taken together, these results demonstrate that the injection of WT M2 macrophages is necessary and sufficient to reverse the bleomycin induced pulmonary fibrosis defect in Rac2-/-. The results strongly demonstrate that the process is macrophage autonomous and the data suggest that it is likely to be dependent on some component of the regulation of the pro-fibrotic M2 macrophage transcriptome within the lung microenvironment.

### Increased expression of alternatively activated M2 genes in BALs isolated from patients with IPF vs controls

**Fig 7:**
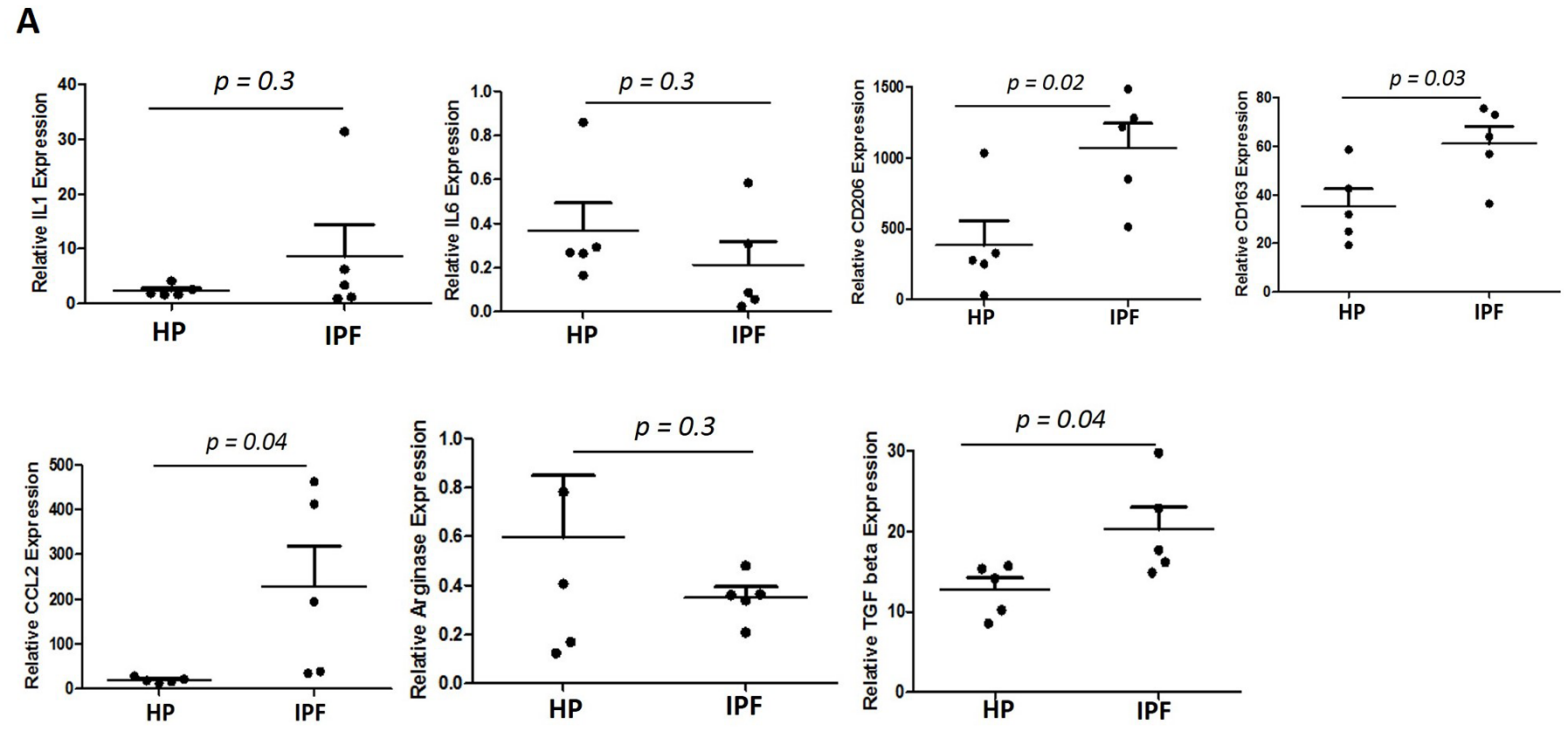
Increased expression of alternatively activated M2 genes in BALs isolated from patients with IPF vs controls. mRNA expression of IL1, IL6/, CD206, CD163, CCL2, arginase and TGFβ in the BALs isolated from human hypersensitive pneumonitis (n = 5) vs. IPF patients (n = 5). Graphs represent mean ± SEM with n = 5. *p ≤ 0.05, **p ≤ 0.01, ***p ≤ 0.001 when compared with HP control.

In order to determine the degree to which the genes involved in alternative activation of M2 macrophages in our mouse model are similar to the human IPF disease setting, we performed RTPCR on human BAL samples isolated from IPF patients compared to disease controls (Fig 7). We performed qPCR on BAL pellets obtained from IPF patients, analyzed for IL1, IL6, CD206, CD163, CCL2, arginase and TGFβ. Our results suggest that expression of CD206, CD163, CCL2 and TGFβ is high in IPF patients as compared to a chronic inflammatory condition that does not progress to fibrosis, hypersensitivity pneumonitis (Fig 7).

## Discussion

Rac2 is a ras-related guanosine triphosphatase expressed mainly in hematopoietic cells, and is a crucial molecule regulating a diversity of endothelial cell integrin and immunoreceptor signaling pathways [22–24]. Previous studies have shown that disruption of the gene encoding Rac2 ameliorated bleomycin induced lung injury [27] and significantly attenuated lung inflammation and injury in an immune complex-mediated acute lung injury model [13]. However, these studies did not describe protection from fibrosis in the Rac2-/- mice or identify the mechanism mediating the Rac2-dependent injury or fibrosis [27]. In the present study, we demonstrated that α_4_β_1_ integrin in macrophages upstream of hematopoietic GTPase Rac2 regulates bleomycin induced fibrosis and M2 macrophage differentiation. Using a murine model of experimental pulmonary fibrosis, we demonstrated that the presence of Rac2, specifically in the macrophages, promotes increased lung injury and collagen deposition. The expression of Rac2 in macrophages skews these cells toward a profibrotic alternatively activated phenotype.

It is well documented that changes in the extracellular matrix (ECM) promote important biological events including inflammation, fibrosis, wound healing, tumor progression and metastasis [36–39]. The present study began with our initial observation that the provisional integrins, α_4_β_1_/α_v_β_3_ induced migration requires a specific isoform of Rac, Rac2, in macrophages and that this pathway regulated the tumor metastasis and alternative activation of M2 macrophages *in vivo* [24]. The α_4_β_1_/α_v_β_3_ integrins are expressed in hematopoietic and endothelial cells [40] and play a key role in inflammation [41–44]. The entry of inflammatory cells and activated fibroblasts into the injured lung depends on the ability of these cell types to interact with components of the ECM, comprised primarily of MMP-digested collagen and fibronectin, the ligands for α_v_β_3_ and α_4_β_1_, respectively. A study by Wang et al has shown that integrin α4 may play an important role in bleomycin induced pulmonary fibrosis, and the use of anti-α4 antibody offers therapeutic antifibrotic potential *in vivo* [39]. The authors have investigated the possible role of the α4 integrin in the pathogenesis of fibrosis by administering an anti-α4 integrin antibody to mice with bleomycin induced lung fibrosis. However, these studies fail to provide a comprehensive picture for how the α_4_β_1_ integrin signals to regulate M2 differentiation and pulmonary fibrosis. Our data demonstrate that α4Y991A k/in mice are defective in bleomycin induced lung injury and collagen deposition. These results are correlated with an impairment in alternative activation of M2 macrophages (Fig 5). Our previous studies have defined a requirement for Rac2 in α_4_β_1_ dependent migration and M2 macrophage differentiation [24]. These data support our hypothesis that the lung fibrosis defect observed in the α4Y991A k/in model is mediated by lack of Rac2 activation downstream of the α_4_β_1_ integrin.

In agreement with a recent study implicating Rac2 in bleomycin induced fibrosis [27], our pulmonary physiology results indicate that late fibrotic lung injury is attenuated in Rac2-/- mice (S1 Fig). We did not observe any differences in lung injury and cellular infiltration at day 3 (S2 Fig), but consistent with the prior report we observed a decrease in infiltration of neutrophils and BAL albumin in the bleomycin challenged Rac2-/- mice at day 7, suggesting a prominent role for Rac2 in transition from early to later phases of bleomycin induced lung injury. We also did not see differences in collagen expression at day 7, but saw increasing collagen expression in WT vs. Rac2-/- mice in the later, fibroproliferative phase. Interestingly, adoptive transfer of M2, but not M1 macrophages in the transition period (day 5) is sufficient to restore the fibrotic response to WT levels (Fig 7). Our observations differ from those of the previous report [27] in that: 1) we did not observe mortality in WT mice up to day 28, 2) our Sirius red staining, αSMA staining and hydroxyproline content (Fig 1) clearly demonstrate a fibrosis defect in Rac2-/- mice at day 28 while the previous report did not report significant differences between WT and Rac2-/- groups at day 21; and 3) we observed a significant increase in TGFβ mRNA in WT samples compared to Rac2-/- after bleomycin (Fig 1C), whereas the prior study showed no qualitative differences in TGFβ immunostaining. The studies use different doses of bleomycin, and slightly different time points and biological measures. As in our study, the authors did not observe any difference in macrophage and lymphocyte numbers in early stages following lung injury in Rac2-/- mice [27]. However, the previous study did not assess macrophage polarization. The defect in infiltration of neutrophils in Rac2-/- mice at day 7 could be an important component in the progression of disease from early to later stages of bleomycin induced lung injury, but the inability of Rac2-/- to promote alternative activation of macrophages in the setting of bleomycin induced injury is a novel and important element of this report. The finding that adoptive transfer of M2, but not M1 macrophages in this time frame is sufficient to restore progression to fibrosis suggests that M2 macrophage polarization, rather than neutrophilic infiltration and vascular leak, is a more important driver of later fibrosis and pulmonary pathology.

Similar to the previous report [27] we found elevated MMP9 levels in macrophages isolated from WT bleomycin mice compared to Rac2-/- bleomycin samples (data not shown). In our study, we observed no disruption of lung architecture on day 3 and day 7 of bleomycin challenge in both WT and Rac2-/- mice, however there is massive infiltration of leukocytes observed on day 7 in both genotypes. Interestingly, we observed increase in expression of inflammatory M1 macrophages in both genotypes on day 3 and day 7 (Fig 3B), with increase in M2 macrophage phenotype in bleomycin challenged WT mice on day 7 (Fig 4C). Most notably, we found a defect in alternative activation of M2 macrophages in Rac2-/- mice (Fig 2) in the late phase of lung injury on day 28. M2 macrophages have been implicated in the pathophysiology of bleomycin-induced injury in murine models [14, 18, 20, 21, 45]. Our previous microarray and genomic studies conducted on BMDMs isolated from Rac2-/- mice provide evidence that Rac2 controls expression of genes related to M2 macrophage differentiation [24].

Our studies suggest that Rac2 provides the signaling specificity to drive the M2 macrophage phenotype under conditions of inflammation where macrophages are interacting with the provisional extracellular matrix and this pathway is required for the development of lung fibrosis. Our data indicate that expression of Rac2 skews lung macrophages toward an alternatively activated profibrotic phenotype, which promotes collagen production, leading to the progression of experimental pulmonary fibrosis. Moreover, we found a significant increase in the expression of CD206 and CD163, typical M2 macrophage markers in human IPF patient samples, suggesting that the study conducted on our mouse model may parallel certain pathophysiologic processes involved in IPF. In conclusion, our findings identify a novel signaling axis which is necessary and sufficient for the control of pro-fibrogenic macrophage differentiation and pulmonary fibrosis *in vivo*.

## Supporting information

S1 Fig Rac2 deficiency prevents bleomycin included changes in pulmonary physiology. Figure shows resistance and elastance measured 28 days after intratracheal saline or bleomycin administration to WT and Rac2−/− mice. Graphs represent mean ± SEM with n = 4 samples/group. One-way ANOVA with post-hoc Tukey’s multiple comparison tests, **p ≤ 0.01 and ***p ≤ 0.001 when WT bleomycin treated group was compared to saline treated groups or the Rac2-/- bleomycin treated group.
(TIF)

S2 Fig Rac2 promotes neutrophil infiltration in early phase of bleomycin induced lung injury. **(A-D)** WT and Rac2-/- mice were given an i.t. challenge with bleomycin and BAL samples were quantified for total cell counts (A), macrophages (B), neutrophils (C) and lymphocytes on day 3 and day 7. Graphs represent mean ± SEM with n = 4–5 samples/group. *p ≤ 0.05, **p ≤ 0.01, ***p ≤ 0.001, # not significant.
(TIF)

S3 Fig Rac2 is required for vascular leakage in early phase of bleomycin induced lung injury. Levels of albumin in BAL supernatants of WT and Rac2-/- mice as determined by BCA protein assay. *p ≤ 0.05, **p ≤ 0.01, ***p ≤ 0.001, # not significant (n = 4–5 samples/group).
(TIF)

S4 Fig Quantitative PCR analysis of mRNA for Cola1 on day 7, 14 and 21. Graphs represent mean ± SEM with n = 3. WT and Rac2-/- mice (n = 4–5 mice/group) were given an i.t. challenge with bleomycin or saline, and whole lungs were isolated on day 7, 14 or 28 and used for RNA isolation and Real Time PCR.
(TIF)

S5 Fig Quantitative PCR analysis of mRNA for M1 (A), M2 (B) specific genes in the BMDMs cultured in MCSF and stimulated with 50 ng/ml of LPS or 20 ng/ml of IL4. Graphs represent mean ± SEM with n = 3 samples. *p ≤ 0.05, **p ≤ 0.01, ***p ≤ 0.001 when compared with no stimulation. Experiment was repeated twice with similar results.
(TIF)

## Abbreviations

BMDM: bone marrow derived macrophages grown in MCSF
ECM: extracellular matrix
i.t.: intratracheal
MCSF: macrophage colony stimulating factor
MΘ: macrophage

## References

[1] Meltzer EB, Noble PW. Idiopathic pulmonary fibrosis. Orphanet J Rare Dis. 2008;3:8 Epub 2008/03/28. doi: 10.1186/1750-1172-3-8 . 18366757 10.1186/1750-1172-3-8 PMC2330030

[2] Noble PW, Barkauskas CE, Jiang D. Pulmonary fibrosis: patterns and perpetrators. J Clin Invest. 2012;122(8):2756–62. Epub 2012/08/02. doi: 10.1172/JCI60323 ; 22850886 10.1172/JCI60323 PMC3408732

[3] Tanaka K, Ishihara T, Azuma A, Kudoh S, Ebina M, Nukiwa T, et al Therapeutic effect of lecithinized superoxide dismutase on bleomycin-induced pulmonary fibrosis. Am J Physiol Lung Cell Mol Physiol. 2010;298(3):L348–60. Epub 2009/12/26. doi: 10.1152/ajplung.00289.2009 . 20034962 10.1152/ajplung.00289.2009

[4] Thannickal VJ, Toews GB, White ES, Lynch JP 3rd, Martinez FJ. Mechanisms of pulmonary fibrosis. Annu Rev Med. 2004;55:395–417. Epub 2004/01/30. doi: 10.1146/annurev.med.55.091902.103810 . 14746528 10.1146/annurev.med.55.091902.103810

[5] Crouch E. Pathobiology of pulmonary fibrosis. Am J Physiol. 1990;259(4 Pt 1):L159–84. Epub 1990/10/01. . 2221080 10.1152/ajplung.1990.259.4.L159

[6] Nuovo GJ, Hagood JS, Magro CM, Chin N, Kapil R, Davis L, et al The distribution of immunomodulatory cells in the lungs of patients with idiopathic pulmonary fibrosis. Mod Pathol. 2012;25(3):416–33. Epub 2011/11/01. doi: 10.1038/modpathol.2011.166 . 22037258 10.1038/modpathol.2011.166 PMC3270219

[7] Mora AL, Torres-Gonzalez E, Rojas M, Corredor C, Ritzenthaler J, Xu J, et al Activation of alveolar macrophages via the alternative pathway in herpesvirus-induced lung fibrosis. Am J Respir Cell Mol Biol. 2006;35(4):466–73. Epub 2006/05/20. doi: 10.1165/rcmb.2006-0121OC . 16709958 10.1165/rcmb.2006-0121OC PMC2643265

[8] Willems S, Verleden SE, Vanaudenaerde BM, Wynants M, Dooms C, Yserbyt J, et al Multiplex protein profiling of bronchoalveolar lavage in idiopathic pulmonary fibrosis and hypersensitivity pneumonitis. Ann Thorac Med. 2013;8(1):38–45. Epub 2013/02/27. doi: 10.4103/1817-1737.105718 . 23440593 10.4103/1817-1737.105718 PMC3573557

[9] Mantovani A, Sica A, Sozzani S, Allavena P, Vecchi A, Locati M. The chemokine system in diverse forms of macrophage activation and polarization. Trends Immunol. 2004;25(12):677–86. Epub 2004/11/09. doi: 10.1016/j.it.2004.09.015 . 15530839 10.1016/j.it.2004.09.015

[10] Gordon S. Alternative activation of macrophages. Nat Rev Immunol. 2003;3(1):23–35. Epub 2003/01/04. doi: 10.1038/nri978 . 12511873 10.1038/nri978

[11] Mosser DM, Edwards JP. Exploring the full spectrum of macrophage activation. Nat Rev Immunol. 2008;8(12):958–69. Epub 2008/11/26. doi: 10.1038/nri2448 . 19029990 10.1038/nri2448 PMC2724991

[12] Geissmann F, Gordon S, Hume DA, Mowat AM, Randolph GJ. Unravelling mononuclear phagocyte heterogeneity. Nat Rev Immunol. 2010;10(6):453–60. Epub 2010/05/15. doi: 10.1038/nri2784 . 20467425 10.1038/nri2784 PMC3032581

[13] Sun L, Louie MC, Vannella KM, Wilke CA, LeVine AM, Moore BB, et al New concepts of IL-10-induced lung fibrosis: fibrocyte recruitment and M2 activation in a CCL2/CCR2 axis. Am J Physiol Lung Cell Mol Physiol. 2011;300(3):L341–53. Epub 2010/12/07. doi: 10.1152/ajplung.00122.2010 . 21131395 10.1152/ajplung.00122.2010 PMC3064283

[14] Gibbons MA, MacKinnon AC, Ramachandran P, Dhaliwal K, Duffin R, Phythian-Adams AT, et al Ly6Chi monocytes direct alternatively activated profibrotic macrophage regulation of lung fibrosis. Am J Respir Crit Care Med. 2011;184(5):569–81. Epub 2011/06/18. doi: 10.1164/rccm.201010-1719OC . 21680953 10.1164/rccm.201010-1719OC

[15] Murray LA, Chen Q, Kramer MS, Hesson DP, Argentieri RL, Peng X, et al TGF-beta driven lung fibrosis is macrophage dependent and blocked by Serum amyloid P. Int J Biochem Cell Biol. 2011;43(1):154–62. Epub 2010/11/04. doi: 10.1016/j.biocel.2010.10.013 . 21044893 10.1016/j.biocel.2010.10.013

[16] Stahl M, Schupp J, Jager B, Schmid M, Zissel G, Muller-Quernheim J, et al Lung collagens perpetuate pulmonary fibrosis via CD204 and M2 macrophage activation. PLoS One. 2013;8(11):e81382 Epub 2013/11/28. doi: 10.1371/journal.pone.0081382 . 24278429 10.1371/journal.pone.0081382 PMC3835428

[17] Collard HR, Moore BB, Flaherty KR, Brown KK, Kaner RJ, King TE Jr., et al Acute exacerbations of idiopathic pulmonary fibrosis. Am J Respir Crit Care Med. 2007;176(7):636–43. Epub 2007/06/23. doi: 10.1164/rccm.200703-463PP . 17585107 10.1164/rccm.200703-463PP PMC2094133

[18] Tao B, Jin W, Xu J, Liang Z, Yao J, Zhang Y, et al Myeloid-specific disruption of tyrosine phosphatase Shp2 promotes alternative activation of macrophages and predisposes mice to pulmonary fibrosis. J Immunol. 2014;193(6):2801–11. Epub 2014/08/17. doi: 10.4049/jimmunol.1303463 . 25127857 10.4049/jimmunol.1303463

[19] Ballinger MN, Newstead MW, Zeng X, Bhan U, Mo XM, Kunkel SL, et al IRAK-M promotes alternative macrophage activation and fibroproliferation in bleomycin-induced lung injury. J Immunol. 2015;194(4):1894–904. Epub 2015/01/18. doi: 10.4049/jimmunol.1402377 . 25595781 10.4049/jimmunol.1402377 PMC4384172

[20] Gharib SA, Johnston LK, Huizar I, Birkland TP, Hanson J, Wang Y, et al MMP28 promotes macrophage polarization toward M2 cells and augments pulmonary fibrosis. J Leukoc Biol. 2014;95(1):9–18. Epub 2013/08/22. doi: 10.1189/jlb.1112587 . 23964118 10.1189/jlb.1112587 PMC3868192

[21] Trujillo G, O'Connor EC, Kunkel SL, Hogaboam CM. A novel mechanism for CCR4 in the regulation of macrophage activation in bleomycin-induced pulmonary fibrosis. Am J Pathol. 2008;172(5):1209–21. Epub 2008/04/12. doi: 10.2353/ajpath.2008.070832 . 18403600 10.2353/ajpath.2008.070832 PMC2329831

[22] De P, Peng Q, Traktuev DO, Li W, Yoder MC, March KL, et al Expression of RAC2 in endothelial cells is required for the postnatal neovascular response. Exp Cell Res. 2009;315(2):248–63. Epub 2009/01/06. . 19123268 10.1016/j.yexcr.2008.10.003 PMC2767303

[23] Pradip D, Peng X, Durden DL. Rac2 specificity in macrophage integrin signaling: potential role for Syk kinase. J Biol Chem. 2003;278(43):41661–9. Epub 2003/08/15. doi: 10.1074/jbc.M306491200 . 12917394 10.1074/jbc.M306491200

[24] Joshi S, Singh AR, Zulcic M, Bao L, Messer K, Ideker T, et al Rac2 controls tumor growth, metastasis and M1-M2 macrophage differentiation in vivo. PLoS One. 2014;9(4):e95893 Epub 2014/04/29. doi: 10.1371/journal.pone.0095893 . 24770346 10.1371/journal.pone.0095893 PMC4000195

[25] Dooley JL, Abdel-Latif D, St Laurent CD, Puttagunta L, Befus D, Lacy P. Regulation of inflammation by Rac2 in immune complex-mediated acute lung injury. Am J Physiol Lung Cell Mol Physiol. 2009;297(6):L1091–102. Epub 2009/10/06. doi: 10.1152/ajplung.90471.2008 . 19801448 10.1152/ajplung.90471.2008 PMC2793190

[26] Yao HY, Chen L, Xu C, Wang J, Chen J, Xie QM, et al Inhibition of Rac activity alleviates lipopolysaccharide-induced acute pulmonary injury in mice. Biochim Biophys Acta. 2011;1810(7):666–74. Epub 2011/04/23. doi: 10.1016/j.bbagen.2011.03.020 . 21511011 10.1016/j.bbagen.2011.03.020

[27] Arizmendi N, Puttagunta L, Chung KL, Davidson C, Rey-Parra J, Chao DV, et al Rac2 is involved in bleomycin-induced lung inflammation leading to pulmonary fibrosis. Respir Res. 2014;15:71 Epub 2014/06/28. doi: 10.1186/1465-9921-15-71 . 24970330 10.1186/1465-9921-15-71 PMC4082672

[28] Feral CC, Rose DM, Han J, Fox N, Silverman GJ, Kaushansky K, et al Blocking the alpha 4 integrin-paxillin interaction selectively impairs mononuclear leukocyte recruitment to an inflammatory site. J Clin Invest. 2006;116(3):715–23. Epub 2006/02/14. doi: 10.1172/JCI26091 . 16470243 10.1172/JCI26091 PMC1361348

[29] Patel AS, Lin L, Geyer A, Haspel JA, An CH, Cao J, et al Autophagy in idiopathic pulmonary fibrosis. PLoS One. 2012;7(7):e41394 Epub 2012/07/21. doi: 10.1371/journal.pone.0041394 . 22815997 10.1371/journal.pone.0041394 PMC3399849

[30] Buendia-Roldan I, Ruiz V, Sierra P, Montes E, Ramirez R, Vega A, et al Increased Expression of CC16 in Patients with Idiopathic Pulmonary Fibrosis. PLoS One. 2016;11(12):e0168552 Epub 2016/12/16. doi: 10.1371/journal.pone.0168552 . 27977812 10.1371/journal.pone.0168552 PMC5158056

[31] Roberts AW, Kim C, Zhen L, Lowe JB, Kapur R, Petryniak B, et al Deficiency of the hematopoietic cell-specific Rho family GTPase Rac2 is characterized by abnormalities in neutrophil function and host defense. Immunity. 1999;10(2):183–96. Epub 1999/03/11. . 10072071 10.1016/s1074-7613(00)80019-9

[32] Kim C, Dinauer MC. Rac2 is an essential regulator of neutrophil nicotinamide adenine dinucleotide phosphate oxidase activation in response to specific signaling pathways. J Immunol. 2001;166(2):1223–32. Epub 2001/01/06. . 11145705 10.4049/jimmunol.166.2.1223

[33] Moore BB, Hogaboam CM. Murine models of pulmonary fibrosis. Am J Physiol Lung Cell Mol Physiol. 2008;294(2):L152–60. Epub 2007/11/13. doi: 10.1152/ajplung.00313.2007 . 17993587 10.1152/ajplung.00313.2007

[34] Ware LB, Matthay MA. The acute respiratory distress syndrome. N Engl J Med. 2000;342(18):1334–49. Epub 2000/05/04. doi: 10.1056/NEJM200005043421806 . 10793167 10.1056/NEJM200005043421806

[35] Diekmann D, Nobes CD, Burbelo PD, Abo A, Hall A. Rac GTPase interacts with GAPs and target proteins through multiple effector sites. EMBO J. 1995;14(21):5297–305. Epub 1995/11/01. . 7489719 10.1002/j.1460-2075.1995.tb00214.x PMC394639

[36] Cox TR, Erler JT. Remodeling and homeostasis of the extracellular matrix: implications for fibrotic diseases and cancer. Dis Model Mech. 2011;4(2):165–78. Epub 2011/02/18. doi: 10.1242/dmm.004077 . 21324931 10.1242/dmm.004077 PMC3046088

[37] Kass L, Erler JT, Dembo M, Weaver VM. Mammary epithelial cell: influence of extracellular matrix composition and organization during development and tumorigenesis. Int J Biochem Cell Biol. 2007;39(11):1987–94. Epub 2007/08/28. doi: 10.1016/j.biocel.2007.06.025 . 17719831 10.1016/j.biocel.2007.06.025 PMC2658720

[38] Butcher DT, Alliston T, Weaver VM. A tense situation: forcing tumour progression. Nat Rev Cancer. 2009;9(2):108–22. Epub 2009/01/24. doi: 10.1038/nrc2544 . 19165226 10.1038/nrc2544 PMC2649117

[39] Wang Q, Wang Y, Hyde DM, Gotwals PJ, Lobb RR, Ryan ST, et al Effect of antibody against integrin alpha4 on bleomycin-induced pulmonary fibrosis in mice. Biochem Pharmacol. 2000;60(12):1949–58. Epub 2000/12/08. . 11108812 10.1016/s0006-2952(00)00491-3

[40] Avraamides CJ, Garmy-Susini B, Varner JA. Integrins in angiogenesis and lymphangiogenesis. Nat Rev Cancer. 2008;8(8):604–17. Epub 2008/05/24. doi: 10.1038/nrc2353 . 18497750 10.1038/nrc2353 PMC2577722

[41] Lobb RR, Hemler ME. The pathophysiologic role of alpha 4 integrins in vivo. J Clin Invest. 1994;94(5):1722–8. Epub 1994/11/01. doi: 10.1172/JCI117519 . 7525645 10.1172/JCI117519 PMC294562

[42] Rose DM, Alon R, Ginsberg MH. Integrin modulation and signaling in leukocyte adhesion and migration. Immunol Rev. 2007;218:126–34. Epub 2007/07/13. doi: 10.1111/j.1600-065X.2007.00536.x . 17624949 10.1111/j.1600-065X.2007.00536.x

[43] Jin H, Su J, Garmy-Susini B, Kleeman J, Varner J. Integrin alpha4beta1 promotes monocyte trafficking and angiogenesis in tumors. Cancer Res. 2006;66(4):2146–52. Epub 2006/02/21. doi: 10.1158/0008-5472.CAN-05-2704 . 16489015 10.1158/0008-5472.CAN-05-2704

[44] Schmid MC, Avraamides CJ, Dippold HC, Franco I, Foubert P, Ellies LG, et al Receptor tyrosine kinases and TLR/IL1Rs unexpectedly activate myeloid cell PI3kgamma, a single convergent point promoting tumor inflammation and progression. Cancer Cell. 2011;19(6):715–27. Epub 2011/06/15. doi: 10.1016/j.ccr.2011.04.016 . 21665146 10.1016/j.ccr.2011.04.016 PMC3144144

[45] Baran CP, Opalek JM, McMaken S, Newland CA, O'Brien JM Jr., Hunter MG, et al Important roles for macrophage colony-stimulating factor, CC chemokine ligand 2, and mononuclear phagocytes in the pathogenesis of pulmonary fibrosis. Am J Respir Crit Care Med. 2007;176(1):78–89. Epub 2007/04/14. doi: 10.1164/rccm.200609-1279OC . 17431224 10.1164/rccm.200609-1279OC PMC2049062

