# Supplementary figures and images for: Rac2 is required for alternative macrophage activation and bleomycin induced pulmonary fibrosis; a macrophage autonomous phenotype
Source: PLoS One. 2017 Aug 17;12(8):e0182851. doi: 10.1371/journal.pone.0182851 (PMC5560537; doi:10.1371/journal.pone.0182851)

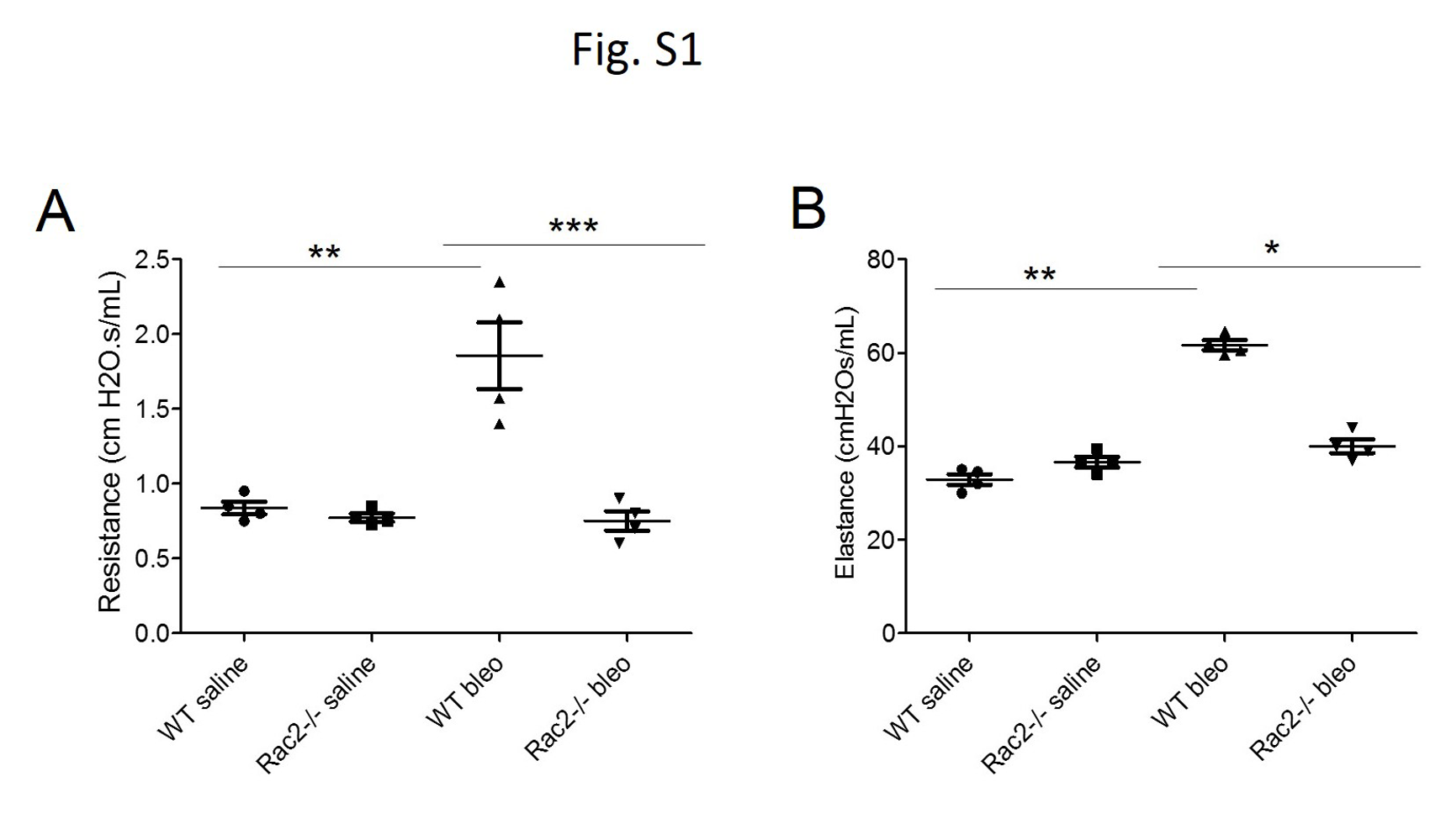

Supplement: S1 Fig — Figure shows resistance and elastance measured 28 days after intratracheal saline or bleomycin administration to WT and Rac2−/− mice. Graphs represent mean ± SEM with n = 4 samples/group. One-way ANOVA with post-hoc Tukey’s multiple comparison tests, **p ≤ 0.01 and ***p ≤ 0.001 when WT bleomycin treated group was compared to saline treated groups or the Rac2-/- bleomycin treated group. (TIF) [file pone.0182851.s001.tif]

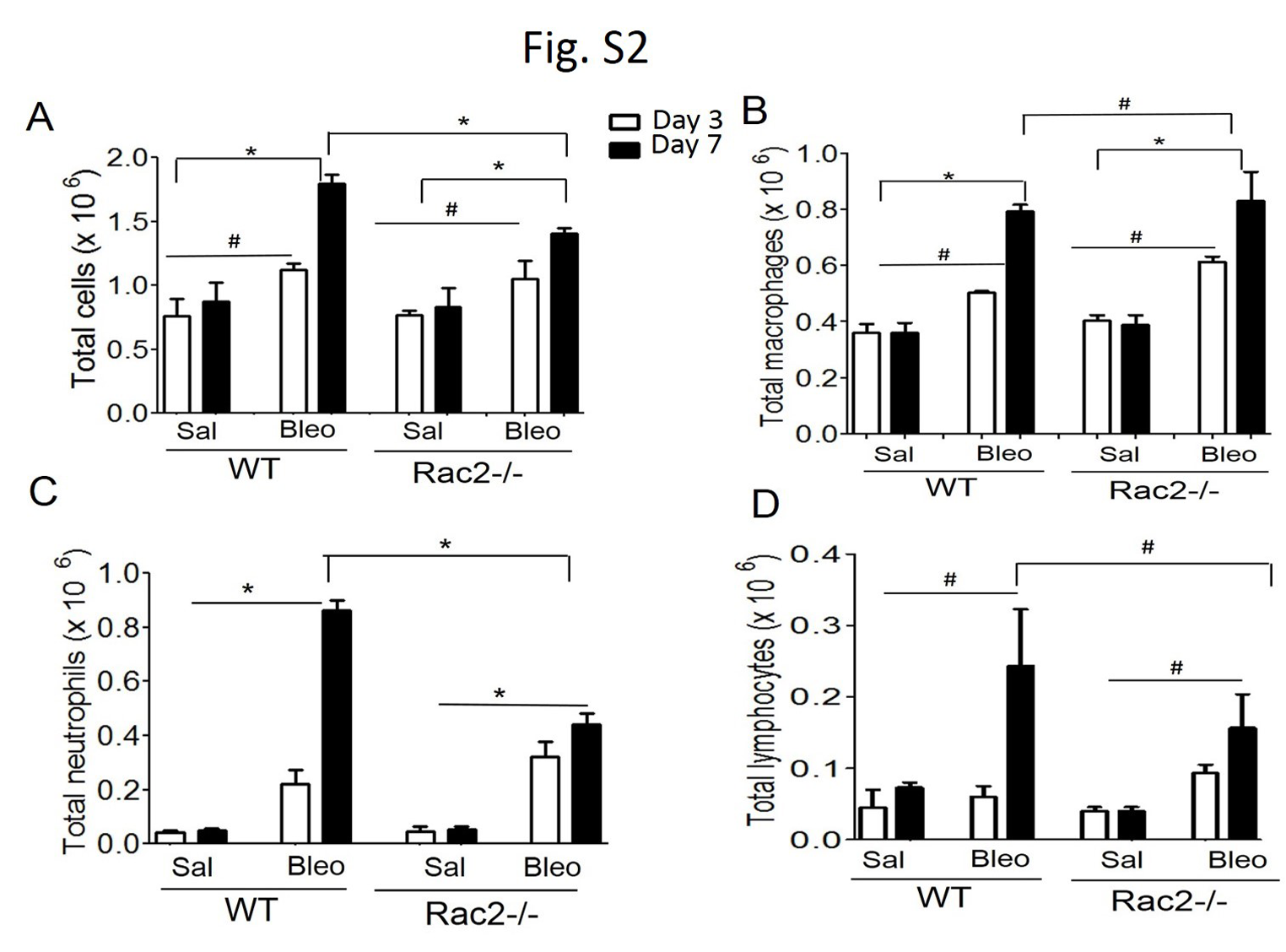

Supplement: S2 Fig — (A-D) WT and Rac2-/- mice were given an i.t. challenge with bleomycin and BAL samples were quantified for total cell counts (A), macrophages (B), neutrophils (C) and lymphocytes on day 3 and day 7. Graphs represent mean ± SEM with n = 4–5 samples/group. *p ≤ 0.05, **p ≤ 0.01, ***p ≤ 0.001, # not significant. (TIF) [file pone.0182851.s002.tif]

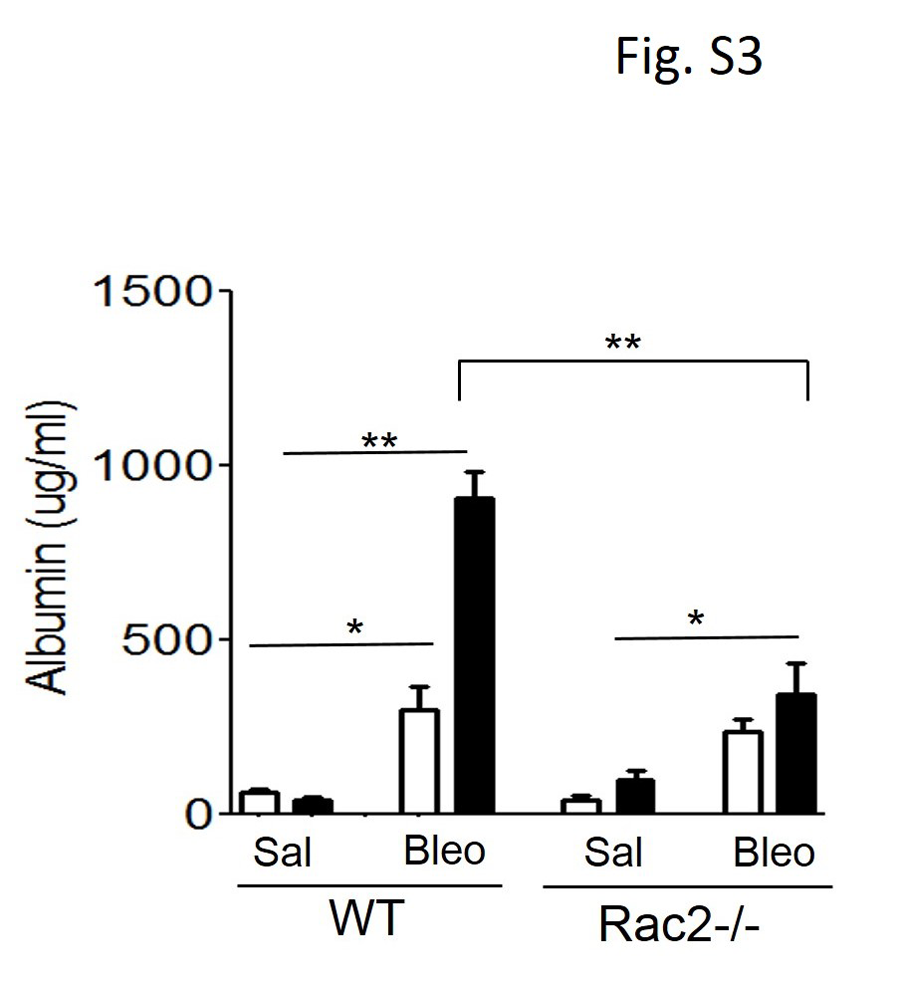

Supplement: S3 Fig — Levels of albumin in BAL supernatants of WT and Rac2-/- mice as determined by BCA protein assay. *p ≤ 0.05, **p ≤ 0.01, ***p ≤ 0.001, # not significant (n = 4–5 samples/group). (TIF) [file pone.0182851.s003.tif]

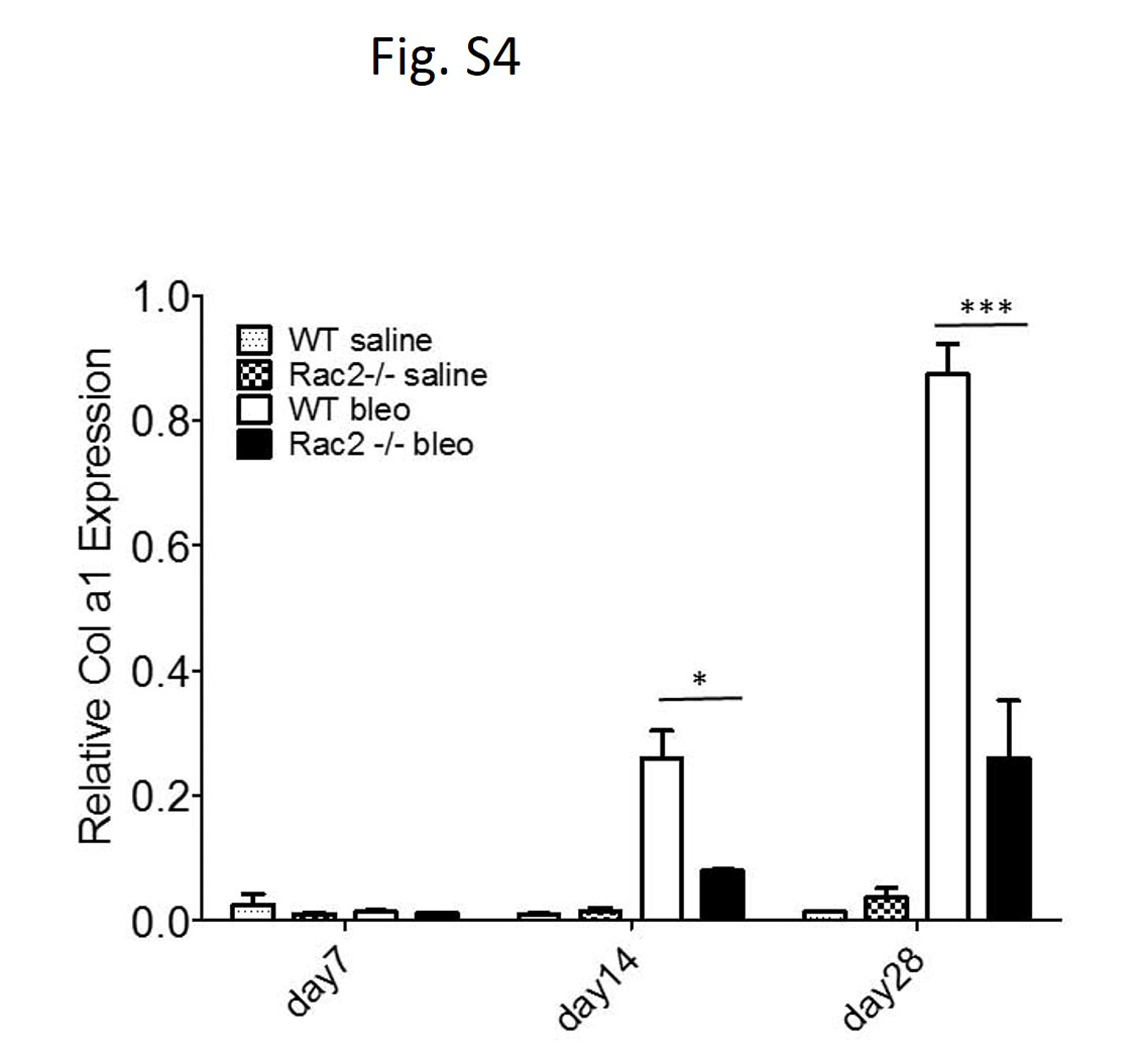

Supplement: S4 Fig — Graphs represent mean ± SEM with n = 3. WT and Rac2-/- mice (n = 4–5 mice/group) were given an i.t. challenge with bleomycin or saline, and whole lungs were isolated on day 7, 14 or 28 and used for RNA isolation and Real Time PCR. (TIF) [file pone.0182851.s004.tif]

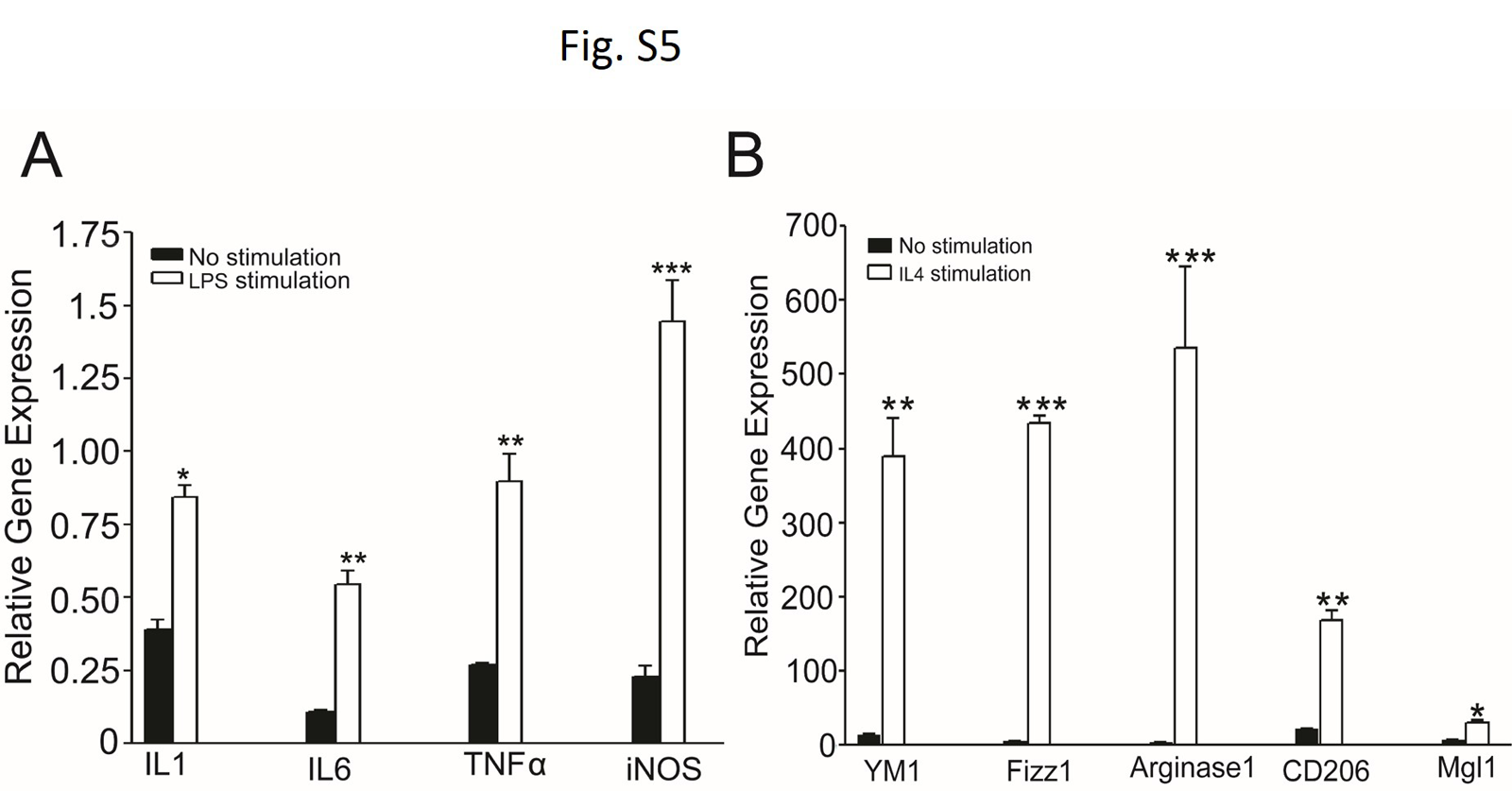

Supplement: S5 Fig — Graphs represent mean ± SEM with n = 3 samples. *p ≤ 0.05, **p ≤ 0.01, ***p ≤ 0.001 when compared with no stimulation. Experiment was repeated twice with similar results. (TIF) [file pone.0182851.s005.tif]
